# Nightly selection of resting sites and group behavior reveal antipredator strategies in giraffe

**DOI:** 10.1002/ece3.6106

**Published:** 2020-02-14

**Authors:** Anna Lena Burger, Julian Fennessy, Stephanie Fennessy, Paul W. Dierkes

**Affiliations:** ^1^ Bioscience Education and Zoo Biology Goethe University Frankfurt Frankfurt am Main Germany; ^2^ Giraffe Conservation Foundation Windhoek Namibia; ^3^ School of Biological, Earth and Environmental Sciences University of New South Wales Sydney NSW Australia

**Keywords:** ecology of fear, giraffe, guarding behavior, nocturnal behavior, predation risk, resting site

## Abstract

This study presents the first findings on nocturnal behavior patterns of wild Angolan giraffe. We characterized their nocturnal behavior and analyzed the influence of ecological factors such as group size, season, and habitat use. Giraffe were observed using night vision systems and thermal imaging cameras on Okapuka Ranch, Namibia. A total of 77 giraffe were observed during 24 nights over two distinct periods—July–August 2016 (dry season) and February–March 2017 (wet season). Photoperiod had a marked influence on their activity and moving behavior. At dusk, giraffe reduced the time spent moving and increasingly lay down and slept at the onset of darkness. Body postures that likely correspond to rapid eye movement (REM) sleep posture (RSP) were observed 15.8 ± 18.3 min after giraffe sat down. Season had a significant effect with longer RSP phases during the dry season (dry: 155.2 ± 191.1 s, *n* = 79; wet: 85.8 ± 94.9 s, *n* = 73). Further analyses of the influence of social behavior patterns did not show an effect of group size on RSP lengths. When a group of giraffe spent time at a specific resting site, several individuals were alert (vigilant) while other group members sat down or took up RSP. Simultaneous RSP events within a group were rarely observed. Resting sites were characterized by single trees or sparse bushes on open areas allowing for good visibility in a relatively sheltered location.

## Graphical abstract

Sleep is essential but it can be a life‐threatening behavioral stage for wildlife. Giraffe, with an array of predators, developed antipredator strategies to survive the night. The tallest land mammal selects “safe” resting sites to take a break during part of the night. Combined with a guarding system that is characterized by some individuals being vigilant (awake) while others are sleeping, these strategies assure the greatest possible security for them from predators.

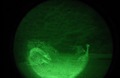

## 1 INTRODUCTION

Prey species regularly adapt and optimize their behavior to increase their chance of survival in a challenging environment while at the same time maximizing available resources. Behavioral decisions aim to decrease the probability of encountering predators and reduce situations of increased vulnerability to predation (Lima & Dill, 1990). However, other behaviors, such as foraging competition or reproduction‐associated behavior, can lead to decisions opposed to predator avoidance. Several studies have shown that the main adaptive behavioral antipredator strategies of African ungulates are related to group size, spatial, and temporal distribution as well as foraging and vigilance behavior (e.g., Crawford et al., 2019; Creel, Schuette, & Christianson, 2014; Davies, Tambling, Kerley, & Asner, 2016; M'soka, Creel, Becker, & Murdoch, 2017; Riginos & Grace, 2008; Thaker et al., 2011; Valeix, Loveridge, et al., 2009). Risky situations cannot always be avoided. For instance, in arid or semi‐arid savannas surface water resources are limited during dry season, often resulting in high levels of animal aggregation (Trash, Theron, & Bothma, 1995) and an increased probability of predator attacks, for example, predominantly by African lion (*Panthera leo*; de Boer et al., 2010). Giraffe (*Giraffa* spp.; Winter, Fennessy, & Janke, 2018) are reported to be particularly vulnerable while drinking due to their body posture with splayed or bent legs (Seeber, Ciofolo, & Ganswindt, 2012; Valeix, Fritz, et al., 2009). As a result, drinking events of giraffe are often preceded by longer scanning periods and increased vigilance (Seeber et al., 2012) as well as an avoidance of waterholes when lions are in the vicinity (Valeix, Fritz, et al., 2009). Sleeping is another behavior which is associated with increased predation risk (Lima, Rattenborg, Lesku, & Amlaner, 2005). There is great variation in terms of sleep duration, occurrence of different sleep states, and behavioral sleeping patterns across species (Campbell & Tobler, 1984; Mignot, 2008; Siegel, 2008). Sleep is generally characterized by distinct behavior patterns such as a species‐specific body posture, rapidly reversible state of immobility, largely reduced motor activity, and an elevated arousal threshold (Joiner, 2016; Siegel, 2005). Sleep in mammals and birds is divided into two main states: rapid eye movement (REM) sleep and non‐REM sleep (Siegel, 2005). Both states are distinguished by changes in brain activity that can be determined by electroencephalograms (EEG). The required duration and state of sleep are thereby often correlated to age, body size, and ecological factors such as the environment diet and safety of the animal's resting site (Ohayon, Carskadon, Guilleminault, & Vitiello, 2004; Siegel, 2005). The presence or absence of predators may have an impact on sleep patterns (Voirin et al., 2014) as well as on nocturnal social group constellation and vigilance behavior (Beauchamp, 2015).

Over the last 30 years, the wild giraffe population has declined by ~30% across its range in Africa and the International Union for Conservation of Nature (IUCN) Red List classified giraffe “vulnerable” (Muller, Bercovitch, et al., 2018). Little is known about giraffe nocturnal activity behaviors in the wild (Baotic, Sicks, & Stoeger, 2015), including their nightly antipredator strategies such as decisions for resting sites, vigilance behavior within the social group, and their sleep behavior. The predicted predation risk for giraffe, while sleeping in open savannah landscapes, is relatively high (Lesku, Roth, Rattenborg, Amlaner, & Lima, 2008). In particular, the rapid eye movement sleep posture (RSP) at ground level makes them likely more vulnerable to predators (Tobler & Schwierin, 1996). Hence, protective strategies such as an increase in group size, a guard system, increase vigilance or choice of resting sites may decrease predation risk during the night. Unitl recently, most research on nocturnal activity and sleep in wild mammals was carried out under experimental conditions, that is, in laboratories or zoological gardens (e.g., Campbell & Tobler, 1984; Duggan, Burn, & Clauss, 2015; Lesku, Roth, et al., 2008). The total sleep time in captive mammals ranges from 3 to 5 hr/day in giraffe (*G. reticulata*) and elephants (*Loxodonta africana*,* Elephas maximus*) to 19 to 20 hr/day in marsupials (e.g., *Lutreolina crassiculata*) and chiroptera (e.g., *Myotis lucifugus*, *Eptesicus fuscus*; Campbell & Tobler, 1984; Tobler & Schwierin, 1996). Captive animals are typically kept under relatively simple, limited and predictable conditions, partly with artificial light, presence of humans (visitors and/or keepers), adequate food supply, relatively fixed social group structures, and in absence of predators. In contrast, natural habitats present a great variety of environmental stimuli and factors influencing nocturnal activity and resting behavior, such as temperature, humidity, light, sound level, structural complexities, conspecific/interspecific interactions, predator risk, and more. As such, observations under experimental or captive conditions are not necessarily generalizable to wild conspecifics (Rattenborg et al., 2017).

Our field study was carried out in Namibia over two seasons (wet and dry season) 2016–2017. Giraffe behavior was recorded during twilight and up to 4 hr after sunset by using night vision cameras and thermal imaging systems. We investigated changes in giraffe activity budget, group size, and RSP behavior as well as selection of resting sites. Increased understanding of giraffe nocturnal behavior will not only help us better understand their behavioral decisions but can also inform effective conservation actions to protect their key habitat and ecological needs.

## 2 METHODS

### 2.1 Study area

Okapuka Ranch, a private farm of ~10,000 ha, was located 30 km north of Windhoek in the Khomas Region, Namibia. Okapuka Ranch is a tourism game farm with game drives occurring. No form of livestock or crop production was undertaken on the farm. The core area is fenced by a 2.4 m high game fence to the west and extends into a mountain range to the east. The farm is a typical arid savannah with mixed woodland‐grassland ecosystem. Natural and artificial waterholes provide permanent access to water for all wildlife on the farm. In addition to Angolan giraffe (*G. giraffa angolensis*), wildlife on the ranch included white rhinoceros (*Ceratotherium simum*) and common African antelope species, for example, common waterbuck (*Kobus ellipsiprymnus*), impala (*Aepyceros melampus*), black and common wildebeest (*Connocheates gnou*, *C. taurinus*), gemsbok (*Oryx gazella*), and greater kudu (*Tragelaphus strepsiceros*). Predators such as leopard (*Panthera pardus*) and cheetah (*Acinonyx jubatus*) were also present. Lions were not naturally present with except for one lioness kept in a separate fenced enclosure.

### 2.2 Behavioral states and resting sites

**Figure 1:**
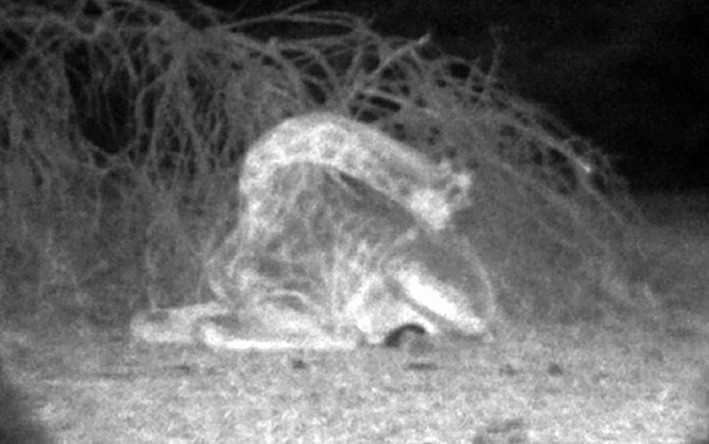
Sleeping posture: During RSP, Angolan giraffe on Okapuka Ranch, Namibia exhibited a characteristic posture bending the neck backwards and resting the head on the flank of a rear leg. Short spontaneous movements of the ears, eyes, or neck were observed with the night vision camera system while they were in RSP

To analyze the nocturnal activity budget of giraffe, we clustered their behavior into active, lying position (resting) and REM sleep posture. An animal was considered active when it did not show a behavior categorized as resting or REM sleep posture. A giraffe was considered resting when it was observed in sternal recumbency on the ground with the abdomen or flank folded under and slightly displaced to the side, and the neck and head erect or slightly bent (Seeber et al., 2012). REM sleep posture was considered when an animal spent more than 10 s lying on the ground, bending its neck backwards and resting its head on the flank/ground (Seeber et al., 2012; Tobler & Schwierin, 1996; see Figure 1). As this specific behavioral state is associated with an enhanced vulnerability, we added special focus on the occurrence and frequency of this behavioral state. During 14 nights, 50 giraffe were observed in RSP. Targeted resting sites were chosen during dusk which equate to times of potential high predator activity and as such examined more closely. The sites were revised the following day and giraffe body imprints were observed on the sandy ground. The size, structure, and surrounding vegetation of the resting sites were recorded. The size of the body imprints helped ascertain the animal's age, that is, adult or calf. The location of the imprints in relation to the surrounding vegetation, other body imprints, and footprints enabled one to gain a more detailed picture of group constellation and space use at a resting site.

### 2.3 Data collection and analyses

The diurnal and nocturnal behavior of Angolan giraffe was directly observed and continuously recorded from vehicle in two different seasons. Data were collected on 24 nights during the dry season (July–August 2016) and the wet season (February–March 2017). Data collection started two hours before sunset (dry season at 5:30 p.m., wet season at 7:30 p.m.) and continued at least two hours after sunset and when possible until midnight. Due to adverse weather conditions, inaccessible terrain and/or group movement, the recording periods differed between nights. Angolan giraffe were observed and their behavior recorded using a night vision system (ATN, Night Scout VX‐CGTI) and up to four thermal imaging cameras (SEEK CompactPro, 320 × 240 pixel) that were attached to a tablet (Apple iPad mini 4) and mounted on a tripod. The thermal imaging cameras had a notably wider angle view and allowed for simultaneous recording of giraffe groups of up to 25 individuals. The recording systems were tested in two zoos with large savannah enclosures (Opel‐Zoo Kronberg and Nuremberg Zoo, Germany) prior to their field use. To investigate the observer's influence on the giraffe's behavior, we tested whether wild giraffe were disturbed by the position of the observer in relation to the observed giraffe, different types of light sources (red‐ and infrared‐light) and noise (hand clapping, human voices, music) (Dagg, 2014; Veasey, Waran, & Young, 1996). Light sources and noise were not observed to cause a notable change in behavior. Similar to other studies on different Artiodactyl, giraffe were less sensitive to the observer when the observer remained in the vehicle (Stankowich, 2008). To minimize disturbance, all observation activities were conducted from within the vehicle. The recording distance between the observer and giraffe depended strongly on vegetation and terrain with an average distance between 40 and 60 m. Giraffe groups were located approximately two hours before sunset and followed until they reached their first resting site for the night. The recording system was then set up, and the behavior of all visible individuals was recorded. As it was difficult to determine a giraffe's age and sex using the thermal image, giraffe were not categorized for behavioral analysis. Additionally, as individual recognition was also not possible using the thermal image, there is a high probability that some individuals were recorded several times during the observation period. In total, 152 RSP events of 50 individuals were recorded (dry season: *n* = 79, 19 individuals; wet season: *n* = 73, 31 individuals). To analyze the influence of social behavior on RSP, we tested whether group size correlated with RSP length. Furthermore, a Wilcoxon test was used to test whether RSP event duration varied seasonally. Statistical analyses were performed in SPSS (v. 24).

## 3 RESULTS

### 3.1 Group behavior and resting sites

**Figure 2:**
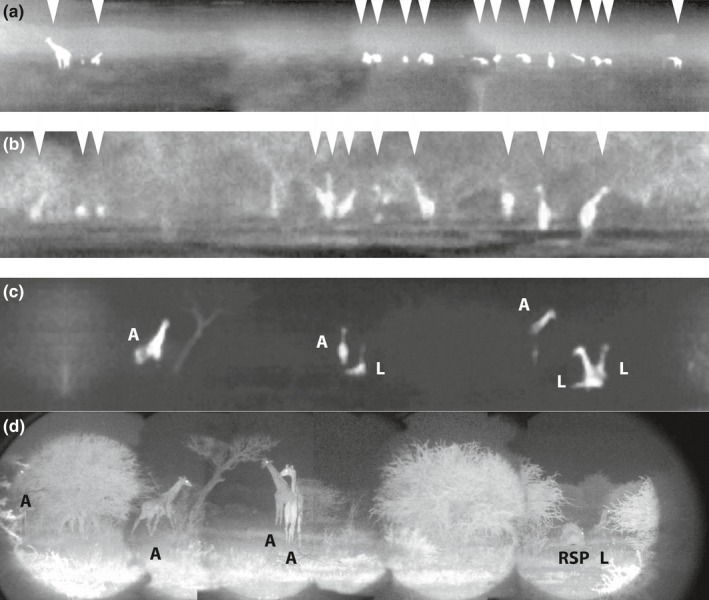
Night panorama: (a) Group movement at dusk (compilation of three thermal images at the same time). (b) Another group stopping in an area with denser vegetation. All individuals in a and b are marked by arrows. In c and d, both pictures were recorded in the same location. (c) The thermal imaging camera with the greater angle view allowed for the recording of several animals at one time including animals partially obscured by vegetation. (d) The night vision camera system allowed for a more detailed picture analysis. Unfortunately, the night vision camera system had a restricted camera angle. Hence, the panorama scan is comprised of six single images. *Note:* All three analyzed activities are shown: active (A), lying (L), and rapid eye movement sleep posture (RSP)

**Figure 3:**
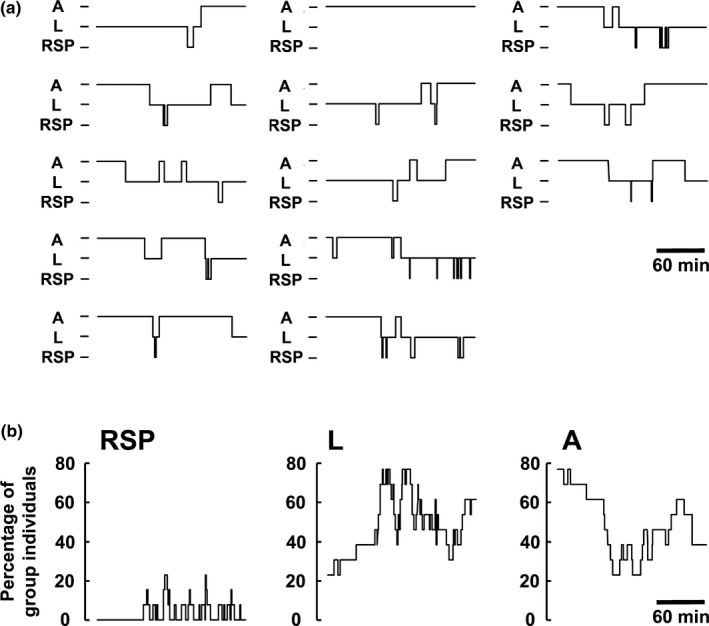
Safeguard system: (a) Behavioral profile of 13 individual Angolan giraffe in a group after sunset (A = active, L = lying, RSP = REM sleep posture) over 3 hr. (b) Activity budget of the giraffe in the group. During the recording time, at least three animals remained vigilant while the others lay down and slept, an indicator of guarding behavior. During the observation phase, RSP events occurred in a periodic sleep‐wake‐cycle whereby simultaneous RSP was only rarely observed by a maximum of three individuals at any one time. First RSP events occurred in this observation 50 min after sunset

During daytime, Angolan giraffe on Okapuka moved in fission–fusion groups of up to 25 individuals, with only adult males observed alone or in single‐sex groups. During dusk, giraffe were generally observed walking in one direction, likely in search for a resting site (Figure 2a). At sunset, the observed group usually stopped and some individuals lay down while others remained active in close proximity (Figure 2b‐d). Giraffe alternated their social role in the group by switching between active, lying and RSP (Figure 3). Most of the active animals were observed feeding or standing. Giraffe often went into RSP after another individual had resumed to lying position from RSP. Simultaneous RSP events within a group were rarely observed with a maximum of three individuals at any one time in RSP (Figure 3). RSP duration did not correlate with group size (Spearman *r*_s_ = −0.035; *p* = .805).

**Figure 4:**
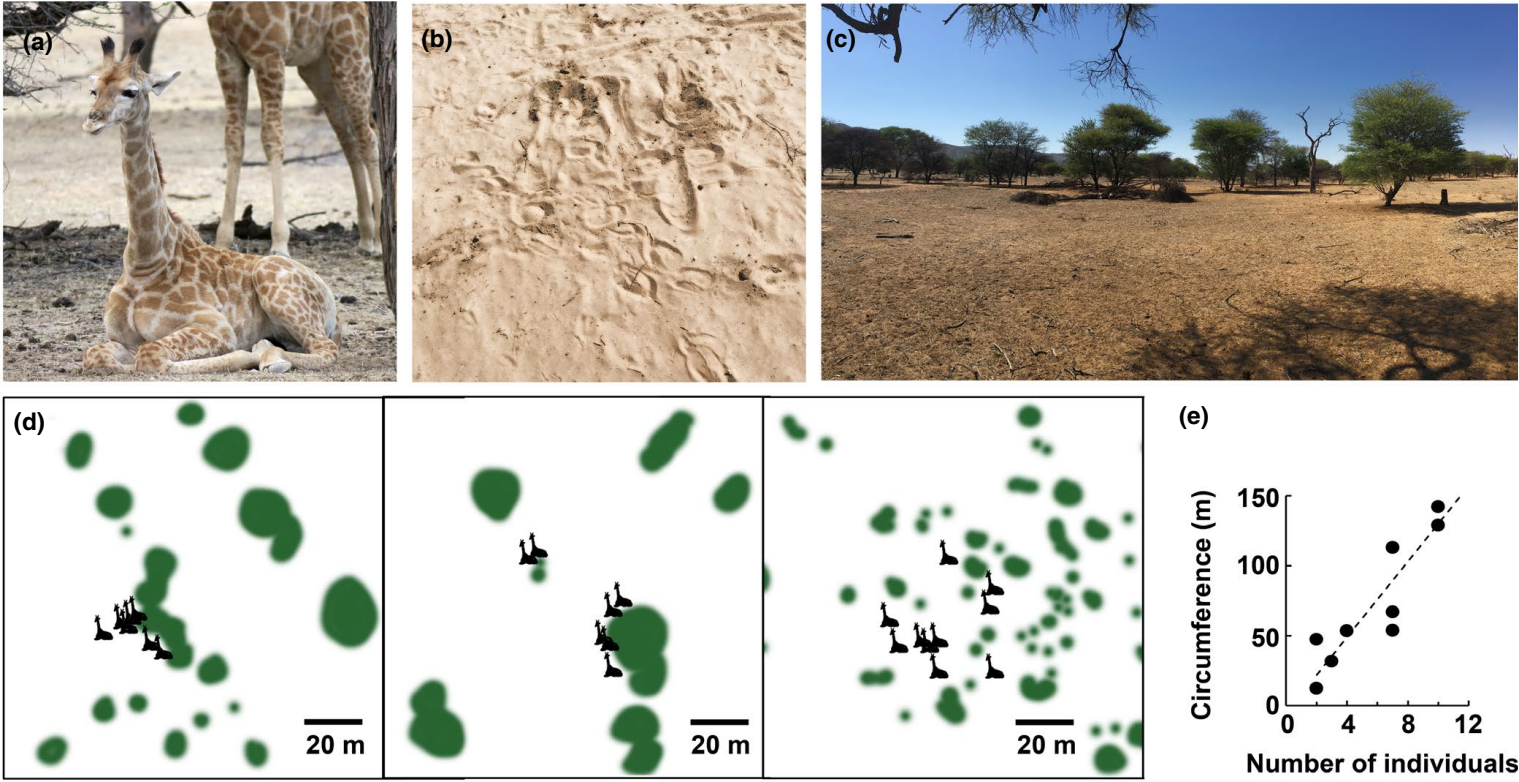
Resting sites: (a) Lying position of an Angolan giraffe on Okapuka Ranch, Namibia. (b) Body imprints of lying animals were easily detectable on sandy ground. (c) View from a typical resting site. (d) Three different resting sites with assigned positions of the individuals. The areas differed in respect to the vegetation density (indicated by the size of green vegetation planes). The resting locations were located near to the existing vegetation. (e) The circumference of resting sites increased linearly with the number of individuals in the group

Resting sites were characterized by either open areas surrounded by a few single trees or by areas with somewhat denser vegetation surrounded by small and sparse bushes smaller in height then a standing giraffe (Figure 4). The spatial positioning of the group members at each resting site differed little during the study. The size of the body imprints of the lying giraffe indicated that older individuals were positioned on the periphery of the resting sites (Figure 4b). Body imprints were usually located in proximity to single trees or bushes and individuals were found within a radius of up to 30 m. These findings were supported by direct night observations. A typical recording situation with six individuals distributed over ~50 m is shown in Figure 2c,d. Giraffe in smaller groups (2–4 individuals) were often observed closer together than giraffe in groups of six or more individuals, where increased mean distance between individuals was observed. In most recordings, the group size varied from 3 to 12 individuals, similar to those observed for Angolan giraffe elsewhere in Namibia (Fennessy, 2004). From dusk until the end of the respective observation period, giraffe stayed relatively close together and did not stray far from the group.

### 3.2 Photoperiod and season

**Figure 5:**
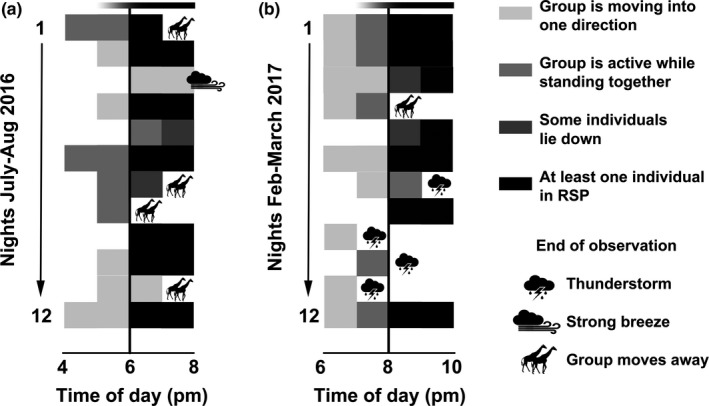
Influence of photoperiod: Activity budgets of Angolan giraffe on Okapuka Ranch, Namibia were observed nightly for two hours before sunset until two hours after sunset. Shortly after sunset, giraffe usually stopped at a preferred site, some of the animals lay down and occasionally RSP events occurred. On some evenings, the observations were canceled due to continuous movements or poor weather conditions. Note that sunset was approximately 2 hr different in time between the dry season 2016 (a) and wet season 2017 (b). Twilight, sunset and darkness are marked with a bar at the top

Photoperiod had a major impact on activity and group dynamics (Figure 5) with giraffe spending less time walking and more time lying after sunset. Comparable behavioral patterns were observed on most nights: The group stopped at one place, and some individuals lay down. After sunset, the observed giraffe spent 25.8% (dry season) and 48.0% (wet season) of their time active, 68.2% (dry season) and 49.9% (wet season) lying, and 6.0% (dry season) and 2.1% (wet season) in RSP. REM sleep posture only occurred after sunset and was observed on 14 of 24 nights (Table 1, Figures 1 and 5) with the first RSP event ~30 min after sunset. In total, 152 RSP events of 50 individuals were recorded (Table 1).

**Table 1:** Summary of Angolan giraffe (*G. g. angolensis*) RSP event data observed during 14 nights in the dry (2016) and wet (2017) season on Okapuka Ranch, Namibia

	Dry season (2016)	Wet season (2017)	Total
Number of nights in which RSP was observed	7	7	14
Total recording time per season [min]	806.8	871.4	1,678.2
Number of observed individuals	28	49	77
Number of individuals in RSP	19	31	50
Mean duration of RSP events ± *SD* [s]	155.2 ± 191.1	85.8 ± 94.9	121.9 ± 156.1
Median of the duration of RSP events [s]	103	35	54.5
Total number of RSP events	79	73	152

Abbreviation: RSP, rapid eye movement sleep posture.

Season had a significant impact on length of RSP events (Wilcoxon test, *p* < .001) with a mean duration of 155.2 ± 191.1 s (*n* = 79, 19 individuals) during dry season (2016), compared to 85.8 ± 94.9 s (*n* = 73, 31 individuals) in the wet season (2017). Giraffe spent 871.0 ± 832.8 s during dry season and 1,284.4 ± 1,328.4 s during wet season lying down immediately before the first RSP sleep event of a resting phase.

## 4 DISCUSSION

### 4.1 Photoperiod and seasonal changes

Our study observed that Angolan giraffe activities on Okapuka Ranch changed regularly at the onset of darkness and the beginning of the night (Figure 5). During twilight, giraffe moved toward suitable resting areas to stop shortly after sunset when some individuals laid down. Natural light seems to be one of the strongest factors influencing sleep onset (Helm et al., 2017; Lesku, Vyssotski, Martinez‐Gonzalez, Wilzeck, & Rattenborg, 2011; Rattenborg et al., 2017), similarly observed in blue tits (*Cyanistes caeruleus*: Steinmeyer, Schielzeth, Mueller, & Kempenaers, 2010) and two sloth species (*Bradypus variegatus; Bradypus pygmaeus*: Voirin et al., 2014). The diel activity of mammals is markedly dependent on natural light, resulting in different rhythm patterns such as nocturnal, diurnal, crepuscular, or cathemeral (Bennie, Duffy, Inger, & Gaston, 2014). The ecology of diel time partitioning is closely connected to predator–prey relationships. It was recently postulated that this temporal partitioning may be a result of reciprocal coevolutionary changes in predation and antipredator behaviors (Wu, Wang, Wang, & Feng, 2018). Hence, spatiotemporal behavioral patterns in mammals have an evolutionary component which may play a role in the “ecology of fear” model (Bleicher, 2017). Within this model, a variety of behavioral strategies are described for prey species to minimize predator encounter rates and the risk of attacks. During the day, larger African ungulates were shown to select an open habitat where the sightlines allow for good visibility of approaching predators. Moreover, group size, foraging, and vigilance behavior increased when predators were present (Creel et al., 2014; Muller, Cuthill, & Harris, 2018; Valeix, Loveridge, et al., 2009). However, minimal data are available for nocturnal behavior, which makes it valuable to analyze the nightly behavioral adaptations observed within the “ecology of fear” model, such as strategies to choose resting sites, to time REM sleep in relation to the behavior of other group members and to regulate length of REM sleep events depending on season.

Our findings clearly show that almost no RSP event occurred during daytime, however, after sunset giraffe started to lie down and RSP events were subsequently observed (Figures 3 and 5). These findings underline the role that change in light may trigger resting behavior in giraffe. The correlation to natural light conditions may be species‐specific, and as such further studies are required. Gravett et al. (2017) showed that light and sunset played no significant role in sleep onset in African elephants, but that environmental conditions (ambient air temperature, relative humidity) had a larger impact. Many ecological factors influence the frequency and duration of sleep. Large herbivores with a high basal metabolic rate, such as African elephants and rhinoceros, have short total sleep times between 3–5 hr/day (Gravett et al., 2017; Santymire, Meyer, & Freeman, 2012), consistent with foraging constraints that limit the time available for sleep (Capellini, Preston, McNamara, Barton, & Nunn, 2010). The recording duration in this study (2–5 hr after sunset) was limited due to adverse weather or terrain conditions, which made it impossible to follow the giraffe throughout the night. Consequently, total resting and sleep time could not be determined.

A study on black rhinoceros did not show a seasonal (dry and wet season) impact on sleep patterns (Santymire et al., 2012), while African elephants’ sleep is affected by ambient air temperature and relative humidity (Gravett et al., 2017). Our study showed that season had a significant effect on the duration of RSP events of giraffe, as also reported for Arabian oryx (*Oryx leucoryx*, Davimes et al., 2018). According to Lima et al. (2005), REM sleep time correlated with the safety of the resting site. Most large prey animals which sleep in relatively unprotected resting sites show less REM sleep (Helm et al., 2017). Our results showed prolonged RSP events during the dry season which may indicate perceived security. Presumably, resting sites were perceived less safe during the wet season due to increased vegetation density associated with a restricted field of vision. However, the seasonal shifts of activity and RSP behavior could also be related to browse quality. We observed that giraffe were searching for specific resting sites characterized by single trees or sparse shrubs allowing for a good view on open areas (Beauchamp, 2015). Further, giraffe were more active and spent less time in RSP during the wet season, in line with other studies on wild giraffe (Dagg, 2014). Several studies on feeding behavior of different wild giraffe species indicate that browse quality is higher (and in turn forage bouts) during the wet season and that giraffe are more likely to migrate during this time to take advantage of new vegetation (Dagg, 2014; Mramba et al., 2017).

### 4.2 Sleep and the risk of predation

The risk of predation is believed to be a marked influencing factor shaping the sleep behavior of prey species (Acerbi & Nunn, 2011; Lima et al., 2005). Moreover, experiments on rats showed that sleep behavior changed after predator encounters, resulting in a reduction of both REM and non‐REM sleep times (Lesku, Bark, et al., 2008). It appears that Angolan giraffe in Okapuka Ranch create a protective situation by optimizing social conditions (guarding system), environmental conditions (resting sites) and an adapted sleep behavior and timing. Similar to captive giraffe (Tobler & Schwierin, 1996), we observed that RSP events were very short (mean duration of RSP events ± *SD*: 121.9 ± 156.1 s) resulting in a fragmented sleep structure (Figure 3). Tobler and Schwierin (1996) noted that the specific body position during REM sleep would likely make giraffe more vulnerable to predation. Additionally, the short RSP events may help avoid vulnerability for giraffe. Recently, Muller, Cantor, et al. (2018), postulated that wild giraffe are able to adapt their behavior to a constantly changing socio‐ecological environment during daytime. Following these results, this study characterized group behavior and typical resting sites of wild giraffe during the night. Predominantly only one or two animals showed simultaneous RSP, while other giraffe were awake and vigilant, a typical characteristic of a guarding system (Caro, 2005; Lima, 1995). During all observation periods, at least one giraffe displayed vigilance and scanned the environment while standing. Vigilance can be distinguished into routine (monitoring of the surroundings during spare time) or induced (costly as foraging is interrupted) vigilance (Beauchamp, 2015). In both cases, vigilance is not only antipredator related but also influenced by habitat structure and intra‐specific social behavior. Cameron and du Toit (2005) showed that wild South African giraffe (*G. g. giraffa*) cows increase vigilance behavior when adult bulls are present in a group and that solitary traveling bulls are less vigilant than those in a group. The presence of calves in a group does not lead to increased scanning behavior (Cameron & du Toit, 2005). Studies on African antelopes (steenbok (*Raphicerus campestris*), oribi (*Ourebia ourebi*), reedbuck (*Redunca arundinum*), impala (*A. melampus*), tsessebe (*Damaliscus lunatus*), blue wildebeest (*Connochaetes taurinus*), sable (*Hippotragus niger*), and buffalo (*Syncerus caffer*) showed for most species that vigilance decreases significantly with increasing group size (Underwood, 1982). Moreover, the influence of cover on vigilance behavior was studied in several mammalian species, and findings show various individual vigilance levels, depending on body size and group size. Wildebeest (*C. taurinus*) for instance scanned less when near dense vegetation but more in open grassland, while impala (*A. melampus*) and springbok (*Antidorcas marsupialis*) increased vigilance behavior when in closed habitats (Bednekoff & Ritter, 1994; Caro, 2005; Scheel, 1993; Underwood, 1982). However, our results did not show a significant effect of group size on RSP event duration. By establishing a guarding system with some group members being vigilant, the other group members in a vulnerable position (lying, RSP) can probably ensure their requirement for REM sleep. Giraffe seem to rely on an active sentinel during their resting and RSP phases of other group members. Besides relying on a sentinel while in a vulnerable position, the choice of the suitable resting site seems to play an important role in antipredator strategies in giraffe. During dusk, giraffe moved purposefully to a resting site where they were observed to lie down close to single trees within open areas or between small and sparse bushes (Figure 4d). At sunset, giraffe mostly stopped walking, and some individuals lay down and slept alternating in RSP. The active animals were recorded as vigilant while feeding or standing. Considering that giraffe spend half of their time feeding (e.g., Fennessy, 2004; Fernandez, Bashaw, Sartor, Bouwens, & Maki, 2008), the need for food intake continues during the night and requires a resting site offering sufficient feeding possibilities. Young and Isbell (1991) reported that the choice of feeding sites for giraffe during the day depends on age, sex, and group constellation. In contrast to female groups without calves or male groups, female groups with calves were observed to feed more likely in open habitats with shorter trees (e.g., Dagg, 2014; Young & Isbell, 1991). This feeding strategy may also occur during the night. On the other hand, places with dense vegetation are considered unsafe resting sites, with an increased risk of a predator attack there. Especially lion use dense savanna vegetation for their ambush‐style hunting strategy (Loarie, Tambling, & Asner, 2013). In conclusion, the continuous behavioral adjustment in choosing resting sites to balance antipredator vigilance, foraging, and resting behavior seems to be an essential task for giraffe.

This study was undertaken in a near‐natural environment. The study area was semifenced and with no free‐roaming lion present. However, understanding the complex predator–prey interplay, predator strategies must naturally be taken into account. Recently, different lion hunting strategies and habitat use were reported to depend on their gender. Male lion generally hunt alone and use dense vegetation to ambush, while female lion often hunt in more socially organized groups in the open (Loarie et al., 2013). The presence and proximity of lion (and other predators) thereby play a crucial role in driving nocturnal movement patterns in herbivores. Zebra (*Equus quagga*) and wildebeest were more active and walked longer distances when lion were near (within 1 km; Traill, Martin, & Owen‐Smith, 2016). As Traill et al. (2016) worked with telemetry data only, no information about the influence of lion presence on vigilance or herd aggregation was gathered. However, Scheel (1993) reported that vigilance in ungulates was affected by herd size, available light and cover as well as by predator presence and activity. Scan rates thereby decreased significantly with increasing herd size (Scheel, 1993). Future studies should focus on the influence of the natural predator of giraffe, the lion. It would be important to compare changes in the nightly vigilance behavior of giraffe and in RSP phases in the presence or absence of lions. Results would then allow to compare nocturnal activity and group behavior of giraffe under high and low predation risk.

### 4.3 Observing nocturnal behavior in the wild—technical aspects and limitations

Studying animal nocturnal behavior in the wild is challenging. Only a few studies have been published on sleeping behavior (Davimes et al., 2018; Gravett et al., 2017; Rattenborg et al., 2017; Santymire et al., 2012; Voirin et al., 2014). Technological innovations, miniaturization and inexpensive, sensitive camera systems now allow for greater long‐term observations and recording of species’ nocturnal behavior in the wild. Video recording and actigraphy are used; however, the chosen method depends on the species’ behavior (Rattenborg et al., 2017). In contrast to southern black rhinos (*Diceros bicornis bicornis*; Santymire et al., 2012), the giraffe groups in this study had no regular resting sites and their location changed nightly. As such, the use of fixed camera traps was not possible and a flexible mobile video recording system was required. Our results indicate that video recording with thermal cameras was an appropriate tool to study wild giraffe in groups of up to 25 individuals. However, a significant limitation of our study was the inability to follow giraffe throughout the entire night. During most nights, the resting periods after sunset were limited to 3–5 hr, where after they continued moving and could not be followed by vehicle predominantly due to habitat inaccessibility. As a consequence, a total nocturnal activity budget or total timing of RSP events could not be determined. However, observations allow for the assumption that wild giraffe show phasic nocturnal activity‐rest rhythms as groups often left the resting site around midnight. Similar nocturnal rhythmicity is reported for captive giraffe (Duggan et al., 2015; Sicks, 2012; Tobler & Schwierin, 1996). For further investigations on the entire nocturnal behavior of wild giraffe, it would be useful to apply GPS tracking units including an accelerometer which have been used successfully for African elephants, sloths (*Bradypus variegatus*; *Bradypus pygmaeus*) and cows (*Bos taurus*) (Fukusawa, Komatsu, Higashiyama, & Oshibe, 2018; Soltis, King, Vollrath, & Douglas‐Hamilton, 2016; Voirin et al., 2014). However, this technology is expensive and unless each individual of a group is equipped with a data logger, analyzing group behavior and group dynamics is impossible (Dominoni, Åkesson, Klaassen, Spoelstra, & Bulla, 2017). Especially for giraffe living in discontinuous bonds (fission–fusion), attaching units to all individuals in any given group is not expedient to investigate social preferences or guarding behavior.

Actigraphy and behavioral recordings comprise uncertainties regarding the differentiation of sleep phases (non‐REM, REM). For instance, a study on wild African elephants revealed a total REM sleep time of only 2.0 hr/day estimated by actigraphy measurements of trunk inactivity without any determination of REM sleep (Gravett et al., 2017). Behavioral recordings may be a better method for assessing REM sleep in wild animals, as parallel recordings of behavioral and EEG data in farm animals have shown (Ruckebusch, 1972; Ternman et al., 2014). For dairy cows, a comparison between electrophysiological sleep and behavioral sleep shows a relatively high correlation of REM sleep phases whereas non‐REM sleep could not reliably be determined by behavioral observations (Ternman et al., 2014). Therefore, behavioral observations may deliver a reliable parameter for determining REM sleep (Santymire et al., 2012) and furthermore REM sleep durations are positively correlated to non‐REM sleep durations in terrestrial mammals (Capellini et al., 2010). In various ruminants, for example, cows, REM sleep is indicated by a specific body posture with a relaxed neck and the head on the flank in a sternal recumbency (Ruckebusch, 1975; Ternman et al., 2014). In horses (*Equus caballus*) or black rhinos, REM sleep similarly occurs in a recumbent position with the head resting on the ground (Santymire et al., 2012; Wöhr, Kalus, Reese, Fuchs, & Erhard, 2016). Studies on captive giraffe and observations in the wild confirmed giraffe to rest in a specific position: lying on the ground, bending their neck backwards, and resting the head on the flank (Seeber et al., 2012; Sicks, 2012; Tobler & Schwierin, 1996). Although there is no physiological evidence to date that giraffe go through REM sleep in this particular body position, the analysis of RSP events can provide useful information about their welfare and behavioral adaptation. However, there is no physiological evidence of REM sleep in giraffe. As Sicks (2012) highlighted in captive giraffe, the frequency of this position correlates significantly with changing environmental and social conditions.

### 4.4 Conservation implication

Our study provides a first detailed analysis of nocturnal behavior of Angolan giraffe in the wild. Information regarding their nightly guarding system, distribution of tasks during resting phases, and the length and timing of RSP events add significant ecological information for understanding behavioral adjustments to decrease their predation risk. These ecological findings translate into valuable conservation implications, in particular how we manage giraffe in the semiwild environment. Especially in today's human‐dominated world, it is imperative to address the human influence on wildlife. Due to declining populations, the recent IUCN Red List status of giraffe was updated to vulnerable (Muller, Bercovitch, et al., 2018). Their decline is mainly caused by human population growth impacts leading to habitat loss and fragmentation, civil unrest, and poaching. Various studies were recently undertaken to determine the influence of human activity on wildlife (e.g., Ciuti et al., 2012; Coetzee & Chwon, 2016; Songhurst, 2017; Stankowich, 2008). Gaynor, Hojnowski, Carter, and Brashares (2018) present a worldwide meta‐analysis on mammals, indicating an alarming increase in nocturnality in mammals caused by human disturbance. Among others, potential disturbing factors are hunting, hiking, urban development, and agriculture. Overall, human presence in wildlife leads to behavioral adaptations in time patterns, social and foraging behavior and habitat use (Gaynor et al., 2018). Against the background of this human–wildlife conflict and in order to counter a further giraffe population decline caused by human intervention, it would be beneficial to learn more about the natural behavior of this animal and their demands on their habitat. As a diurnal animal, the giraffe is dependent on meeting its natural need for rest and sleep during the night. To ensure that this is the case, human night activities should be carefully planned, especially on wildlife farms or in safari areas. Traveling by vehicle should be given preference over walking, and vehicles should stay on the roads as this was reported to have less affect on the behavior of wild animals (Stankowich, 2008; Veasey et al., 1996).

By defining wild giraffe's nocturnal behavior and habitat use, this study seeks to better understand giraffe sleeping behaviors in the wild. Together with other studies, results can increase our understanding of giraffe sleep patterns and the resulting conservation implications will hopefully inform greater protection of giraffe and their habitat throughout Africa.

## CONFLICT OF INTEREST

None declared.

## AUTHORS’ CONTRIBUTIONS

A.L.B and P.W.D. conceived the ideas, designed methodology, collected and analyzed the data, and wrote the manuscript; J.F. and S.F. provided technical advice to the research, supported research activities in Namibia, contributed to the drafts, and gave final approval for publication.

## Data Availability

Data will be publically available from Dryad after publication of this article. Dryad https://doi.org/10.5061/dryad.vdncjsxqr
